# Process Evaluation of an Effective Multifaceted Quality Improvement Intervention to Improve Acute Stroke Care: Unpacking the Success Factors and Challenges

**DOI:** 10.34172/ijhpm.9013

**Published:** 2026-03-10

**Authors:** Tara Purvis, Elizabeth Lynch, Violet Marion, Julie Morrison, Monique F. Kilkenny, Sandy Middleton, Dominique A. Cadilhac

**Affiliations:** ^1^Department of Medicine, School of Clinical Sciences at Monash Health, Monash University, Clayton, VIC, Australia.; ^2^College of Nursing and Health Sciences, Flinders University, Adelaide, SA, Australia.; ^3^Stroke and Critical Care Research, The Florey Institute of Neuroscience and Mental Health, University of Melbourne, Heidelberg, VIC, Australia.; ^4^Nursing Research Institute, St Vincent’s Health Network Sydney, St Vincent’s Hospital Melbourne and Australian Catholic University, Sydney, NSW, Australia.; ^5^School of Nursing Midwifery and Paramedicine, Australian Catholic University, Sydney, NSW, Australia.

**Keywords:** Mixed-Method, Stroke, Facilitation, Australia

## Abstract

**Background:**

The Shared Team Efforts Leading to Adherence Results (STELAR) program is a multifaceted quality improvement intervention directed at hospital clinicians to improve stroke care. In a stepped-wedge cluster trial (N=9 hospitals, Australia), STELAR achieved a 17% improvement in adherence to prioritized clinical indicators (composite outcome). We report the critical success factors and challenges that influenced change in care delivery.

**Methods:**

STELAR included two externally facilitated workshops; (*i*) feedback of national registry data to identify practice gaps and prioritize indicators; (*ii*) barrier assessment and action plan development (2017-2018). Hospitals appointed a site coordinator, and identified local change champions. Two months of remote-support followed to implement action plans. The process evaluation included workshop observations (N=18), document review, satisfaction surveys (N=51), and semi-structured interviews (external facilitator, N=9 clinician site coordinators). Qualitative data were mapped to an implementation framework before inductive thematic analysis. Quantitative data were analysed descriptively, with all data triangulated.

**Results:**

Critical success factors included delivery by knowledge translation experts, external facilitation support/nudging to maintain staff engagement at hospitals, and the use of evidence to self-select prioritized indicators. Involving multidisciplinary staff in action planning and supporting local change champions to lead the implementation fostered capacity building. Reported challenges related to insufficient time when focusing on several prioritized indicators simultaneously within a limited timeframe and having to address strategies that involved working with different clinicians or hospital departments. Staff workload and availability and lack of medical buy-in and management support during the process were wider organizational challenges reported.

**Conclusion:**

Despite the identified challenges imposed by the limited implementation period, STELAR’s multifaceted attributes underpinned the overall strong positive change in quality of stroke care. Implications for wider adoption include involvement of knowledge translation experts with ongoing support for capacity building, and allowing greater time to work through implementation strategies.

## Introduction

**Key Messages**

**Implications for policy makers**

- Invest in credible, standardized data systems: Reliable national clinical registries enable hospitals to identify performance gaps, benchmark care, and monitor progress objectively, forming the foundation for data-driven quality improvement.
- Fund structured facilitation and support: External facilitation and “nudging” from implementation experts enhances engagement, accountability, and change, especially in resource-limited or more regional settings.
- Build local implementation capacity: Empowering hospital staff with practical training in change management and establishing numerous “local clinician champions” strengthens ownership and supports long-term improvement.
- Prioritize realistic, targeted improvement goals aligned with timeframe: Focusing on fewer, feasible indicators with clear actions that can be addressed within a predefined timeframe will improve the likelihood of success. Ensuring strategies address the major “modifiable” barriers identified eg, investing time in building relationships to achieve stakeholder buy-in if engagement with one professional group is suboptimal.
- Enable leadership and system alignment: Visible executive and medical support, adequate resourcing, and alignment with existing organizational priorities and strengthening collaborations beyond the organization are essential for shared learning and embedding and sustaining improvements in stroke care delivery.

**Implications for the public**

Stroke care in hospitals can be complicated, and many patients do not always receive the recommended care, which can affect their recovery. Initiatives that improve how care is delivered are therefore essential. The Shared Team Efforts Leading to Adherence Results (STELAR) program was designed to support hospital staff to improve stroke care. STELAR provided workshops followed by online support over two months to local “change champions.” Results of the original trial showed that hospitals involved in STELAR delivered better stroke care overall, with a 17% improvement in care quality. This research examined why STELAR work so well. Using observations, surveys, and interviews, we found that hospitals were most successful when they focused on a small number of improvements, set realistic goals, built staff skills, and shared responsibility across the team. Allowing more time to put plans into action could support more complex improvements, particularly those involving multiple departments.

Globally, stroke is the second leading cause of death and third leading cause of disability.^1^ Evidence-based clinical practice recommendations for stroke treatment are well-established in many countries and emphasize timely delivery of proven interventions such as thrombolysis, stroke unit care, and coordinated multidisciplinary team management.^2-4^ Despite these guideline recommendations, variation in the quality of stroke care provided in hospitals remains an issue.^5,6^ This contributes to preventable differences in patient outcomes, including increased rates of post-stroke complications, poorer survival, and lower quality of life.^7,8^

 Quality improvement interventions reported to be effective for reducing variation in practice include audit and feedback, reminders, opinion leaders and educational outreach.^9,10^ Having facilitators to enable and assist clinicians, teams or organizations to change and improve clinical practice is also effective.^11^ No single intervention is suitable for all situations. However, there is evidence that combining multiple strategies may be beneficia,^12^ especially when local barriers and facilitators to the care practices are addressed.^9,13^ The ability to routinely review local performance data, such as via a clinical quality registry program, can further strength the effectiveness of stratgeies.^5^ However, it remains unclear which combination of improvement interventions are most effective in guiding evidence-based practice, particularly in the field of stroke.^14,15^

 In 2018, our team completed a stepped-wedge randomized controlled trial of STELAR (Shared Team Efforts Leading to Adherence Results), a co-designed, theory informed, multifaceted quality improvement program directed at hospital clinicians to improve acute stroke care.^16^ The intervention was designed to enhance the use of Australian Stroke Clinical Registry (AuSCR) data^17^ with a focus on providing external knowledge translation support informed by the Promoting Action on Research Implementation in Health Services (PARIHS) framework^18^ through two workshops and support (2 months).^16^ A nominated site coordinator (clinician) was responsible for inviting all multidisciplinary staff involved in stroke care or quality improvement activities at their hospital to take part in the STELAR program. Contextual information including perceived areas of practice gaps, and barriers impacting performance was obtained from a pre-workshop survey. The survey information and local hospital registry data were used in the first workshop to support staff prioritizing 2-3 indicators (out of 10 AuSCR indicators). Action plans were then collaboratively developed through a consensus-based approach, incorporating tailored implementation strategies to address modifiable barriers related to the prioritized clinical indicators. Local “change champions” were appointed to oversee and support the implementation of strategies at their hospital, with 2-months of time-limited remote contact provided by the external facilitator. The intervention was delivered as planned at the cluster level, with a 17% net improvement (range 3%-30%) in the primary composite outcome reported for the intervention period when compared to the control period, including an overall 6% greater adherence for all indicators (range 3% to 10%; *P*< .001).^16^ Based on comparison with prior reviews ^9,10,19^ the results indicated a large effect size. Therefore, describing the critical success factors for what led to this effect size is important to describe. The aim of this article is to describe (1) the critical success factors that influenced practice change and care delivery to support replication and up-scale and spread; and (2) detail the identified challenges, that if overcome, might optimize the intervention further.

## Methods and Materials

 The STELAR trial methods have been published elsewhere.^16^ Additional details related to these methods can be found in Supplementary file 1. For this study, we used data obtained from the process evaluation undertaken in parallel with the STELAR effectiveness trial to understand factors that influenced implementation of the action plans in achieving practice change. Process evaluations are particularly important in multifaceted, multi-centred trials, where the same intervention may be received and then implemented in different ways.^20^ Understanding the factors and contextual influences that may lead to variations in outcomes is important to support replication in the field and future adoption of the quality improvement intervention.^20^

 The program logic of STELAR is depicted in Figure.

**Figure:**
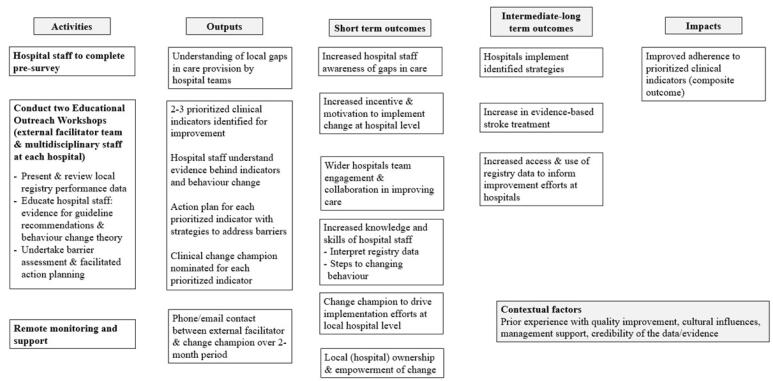
The Shared Team Efforts Leading to Adherence Results Program Logic.

###  Process Evaluation Methods

 The process evaluation design was guided by the framework for designing and reporting process evaluations for cluster-randomized trials outlined by Grant et al.^21^ Accordingly, data were collected about the intervention delivery at each hospital, and the local implementation of changes to practice and care delivery, under the headings of reach, delivery, clinician response and sustainability across both levels. To complement this evaluative structure, the i-PARIHS (Integrated-Promoting Action on Research Implementation in Health Services) framework^22^ provided a theoretical lens to explore *how* and *why* the facilitation intervention and implementation succeeded as well as the challenges encountered. It focused on the innovation (evidence), the roles of recipients, the broader contextual environment in which the intervention was delivered and change was planned, and the facilitation processes supporting both intervention delivery and implementation. This determinant framework was considered fitting as it aligned with the theoretical basis of the STELAR program.

 All components of this process evaluation were summarized and interpreted with consideration of context and in relation to the main trial results. The Consolidated Criteria for Reporting Qualitative research (COREQ)^23^ has been utilized for reporting of the methods and results of the qualitative data presented in this article (Supplementary file 2).

###  Data Collection

 Data were collected both prospectively and retrospectively at various timepoints across all steps to capture information on intervention delivery (July 2017–May 2018) and local implementation (September 2017–September 2018). Sources included direct observations of workshops, document analysis (eg, attendance lists of workshops, support activity log), a post-workshop clinician satisfaction survey, and semi-structured interviews conducted as the end of the support period (Table 1). Contextual information about each hospital was obtained from the pre-workshop survey.

**Table 1:** Data Sources and Collection Methods Related to the Process Evaluation

**Evaluation Domain**	**Research Focus**	**Data Source**	**Type of Data**
Reach	Who received the intervention at each hospital?	Workshop documents	Attendee list from workshops
	Are they representative?	Interviews with site coordinators	Explore challenges to engagement
Intervention delivery (hospital)	What was delivered? Was it as intended? (fidelity, dose)	Direct observations of workshops	Components of the intervention delivered
		Action plan	Action plan completion, and number of processes identified
		Support activity log	Nature and amount of support provided by external facilitator
		Interview with external facilitator	Barriers and enablers to delivery of the intervention
Response to the intervention (hospital staff)	How did the participants respond to the intervention.	Post-workshop Satisfaction survey (end of workshop 2)	Closed and open text responses on satisfaction of the intervention to date, and areas for improvement
		Interviews with site coordinators	Identify which aspects of STELAR intervention were most helpful
Implementation (local)	How is the intervention adopted?	Support activity log	Summary of activities/strategies implemented locally
	What was implemented locally.	Interviews with site coordinators and external facilitator	Explore what was implemented locally and barriers/facilitators to this
Contextual influences	Wider context in which the trial is being conducted. Environmental issues that may influence implementation.	Pre-survey responses	Rating scale/open text responses for perceived areas for clinical improvement and barriers affecting delivery of care
		Action plan	Perceived barriers to delivery of care
		Interviews with site coordinators and external facilitator	Explore contextual influences on implementing clinical change
Maintenance	Are the changes likely to be sustained over time? Why, why not?	Interviews with site coordinators and external facilitator	Sustainability of change

Abbreviation: STELAR, Shared Team Efforts Leading to Adherence Results. Based on framework for process evaluations for cluster randomized trials (Grant et al^21^).

####  Intervention Delivery 

 Data informing the intervention delivery were obtained through workshop observations, attendance records, action plans, and a post-workshop survey. All workshops were observed (by TP), audio-recorded with permission, and supported by detailed field notes. Attendance lists were used to document workshop participation, and action plans (Supplementary file 3) were reviewed to assess the number of indicators selected for improvement and the strategies identified.

 A satisfaction survey, developed in collaboration with the research team, comprising both closed and free-text questions was administered at the end of the second workshop to gather feedback on the intervention delivery and suggestions for improvements (Supplementary file 3).

####  Local Implementation 

 Data related to local implementation were drawn from the support activity log and semi-structured interviews. Each hospital’s support activity log, completed by VM during the support period, was reviewed to summarize the number, type, and nature of locally implemented activities (Supplementary file 3).

 Following completion of their respective support period, all clinician site coordinators were invited to participate in a semi-structured interview exploring factors that influenced local implementation, as well as intervention delivery (March 2018–July 2018). The interview guide included open-ended questions about the intervention delivery and facilitators, barriers, and potential improvements to implementing clinical change (Supplementary file 3). In addition, the external facilitator, VM, was interviewed to provide broader insights into overall program delivery and local implementation across hospitals (August 2018).

 All interviews were conducted by author TP (female, Masters in Science, experienced qualitative researcher, with expertise in stroke research and clinical care) via phone/teleconference, with no other project personnel present. TP was independent of intervention delivery but was known to participants from workshop observations. Interviews lasted approximately 60 minutes and were audio recorded.

###  Data Analyses 

 De-identified, transcribed qualitative data were managed using NVivo 12 (QRS International Pty Ltd). No participants chose to review their transcript prior to analysis. A hybrid analytical approach was undertaken.^24^ Two researchers independent of the of the STELAR intervention (TP; EL, female, PhD, experienced qualitative researcher) first deductively mapped interview data to the broad constructs of the i-PARIHS framework followed by inductive coding within each construct. A coding tree outlining themes and sub-themes was iteratively developed through a consensus-building process, in which the two reviewers independently coded three transcripts, discussed discrepancies, clarified definitions, and agreed on final codes. The coding tree was refined and verified by DAC, through cross-checking a sample of coded transcripts to ensure consistency and interpretive accuracy. The finalized coding tree was then used to systematically code all transcripts (TP).^25^ Flexibility was maintained throughout the process, with the coding tree iteratively adjusted to accommodate new themes or insights emerging from later transcripts.^25^ This hybrid approach enabled exploration of both descriptive and interpretive meanings, supporting a richer understanding of the data in relation to the research.^26^ Free-text responses from the post-workshop survey and support activity log were analysed using a similar approach. Quantitative data (eg, attendance, closed survey responses) were summarized descriptively using Stata/SE 15.01 (StataCorp 2017).

 All quantitative and qualitative data from workshop observations, attendance lists, post-workshop surveys, support activity logs and interviews were explored using a multiple triangulation approach, drawing on diverse methods, data sources (eg, hospital staff and VM), and analysts to enhance the depth and credibility of findings.^27^ The triangulation process was iterative and interpretive. It started with separate analyses of each data source, followed by a systematic cross-source comparison to identify areas of convergence, divergence and complementarity and to explore how themes/sub-themes aligned or differed (TP). All data sources were mapped to the i-PARIHS framework to support integrated interpretation and ensure consistency across evaluation domains. Illustrative quotes are provided verbatim.

## Results

###  Characteristics of Participants

 All 18 workshops were observed. A total of 82 staff attended the second workshop, and 51 completed the satisfaction survey (response rate 62%). One site coordinator resigned shortly after the second workshop, and VM continued to support to remaining team members from that hospital. All nine site coordinators and the external facilitator (VM) participated in interviews conducted between March and July 2018 (median 99 days post-support period). All site coordinators were nurses (78%) or allied health clinicians (22%), with the majority working part time (78%). Additional characteristics are summarized in Supplementary file 4.

 In the following sections the main themes and sub-themes that influenced practice change, including success factors and ongoing challenges, are presented according to the i-PARIHS framework constructs (Table 2 and Supplementary file 5). These findings draw on data from all sources and are described with reference to both the intervention delivery and local implementation.

**Table 2:** Summary of the Themes and Subthemes Including the Success Factors (+’ve), Challenges and Areas for Ongoing Improvement (-‘ve) Based on the i-PARIHS Framework

**i-PARIHS Construct & Definition**	Relevant STELAR Program Features*	**Themes and Subthemes**	**+‘ve **	**-‘ve **
*Facilitation (role and process of program delivery)* The way in which the evidence was introduced, and by whom	*Intervention delivery to hospitals: *Involved external facilitation and external process, including facilitated educational outreach workshops, barrier assessments, local consensus process, and virtual support *Local implementation:* local change champions (internal clinical facilitation) were appointed to lead practice change and implementation of agreed evidence-based strategies	**Importance of the research team and external facilitator**		
		● External involvement fostered local engagement	✓	
		● Importance of relationships with local staff	✓	
		● Benefit in online and face-to-face workshops	✓	
		● Nature and value of support offered by the external facilitator	✓	✓
		**Focused resources and education**		
		● Need for structured implementation plan		✓
		● Further behaviour change education		✓
		● Notification of project finalization		✓
		**Role of the internal clinical facilitator**		
		● Site coordinator as local change champion		✓
		● Enabling approach of site coordinator	✓	
*Innovation/Evidence * Sources of knowledge, quality and type of evidence	*Intervention delivery to hospitals: *Multifaceted intervention including use of national stroke registry data (AuSCR) to identify local care gaps related to evidence-based care	**Data driven intervention using existing performance data **		
		● Minimized additional time and resource requirements		
		● Inclusion of broad range of clinical indicators important	✓	
		● Data quality		✓
		● Improved clarity of definitions of AuSCR data	✓	
		**Characteristics of the action plan**		
		● Prioritization of the clinical indicators	✓	
		● Number of prioritized indicators		✓
		● Complex strategies identified		✓
		● System or process change vs. person change	✓	
*Recipients * The individuals involved in implementing change	Multidisciplinary team involved with stroke care or improvement initiatives in nine hospitals in Victoria involved during the *intervention delivery *and* local implementation processes*	**Team involvement**		
		● Engagement of key stakeholders in process	✓	
		● Prioritization of quality improvement in wider staff roles	✓	
		● Limited medical involvement		
		● Staff rotation/unexpected absences/workload		✓
		● Influence of existing relationships		
*Context* Characteristics or quality of the environment that may enable or constrain implementation; consideration to local, hospital level, as well as wider external context	Local setting related to hospital unit, ward or organization, and wider health system including networks (influenced both *intervention delivery and local implementation processes)*	**Organizational context**		
		● Alignment with hospital priorities	✓	
		● Competing organizational demands		✓
		● Change fatigue		✓
		**Management & leadership support**		
		● Limited medical buy-in and managerial support (workshops, implementation activities)		✓
		● Limited resource allocation		✓
		● Collaboration with hospital quality improvement team	✓	
		**Inter-organisational collaborations**		
		● Support from Regional Stroke Coordinator Network	✓	
		● Sharing of resources		
*Successful implementation* Achievement of agreement of implementation/project goals. Uptake and embedding of the innovation/evidence in practice	*Intervention delivery to hospitals: *External process - delivery of the workshops as planned to hospitals *Local implementation: * Internal process – implementation of agreed strategies outlined in the action plans, to ultimately lead to practice change related to identified indicators	**Implementation versus practice change**		
		● Longer time required to implement strategies to achieve desired behaviour change		✓
		● Wider benefits to stroke care	✓	
		● Consideration to ongoing improvement	✓	

Abbreviations: i-PARIHS, Integrated-Promoting Action on Research Implementation in Health Services; AuSCR, Australian Stroke Clinical Registry; +’ve, critical success factor;-‘ve,barriers, challenge or improvement areas. * Considering intervention delivery (to hospital clinicians) and implementation (locally).^20^ Adapted from Harvey et al.^22^

###  Facilitation

####  Importance of the Research Team and External Facilitator

 Within the post-workshop surveys and interviews, participants frequently reported that having the STELAR program coordinated by an external organization and delivered by facilitators with expertise in implementation science added to the credibility of the program. This also encouraged broad buy-in and engagement from the local hospital staff:

 “*It was all stuff we knew we needed to be doing... but when you’ve got someone external involved there is more impetus to drive it... Without the external involvement, I don’t know whether we would have got much happening*” (Interview, site coordinator, H6).

 “*Discussing issues with multiple professions: ie. to get nursing input and opinion on topics also affecting allied health was VERY beneficial; broke down barriers well!*” (Survey, speech pathologist, H4).

 Overcoming the perception of isolation in regional hospitals was important and was aided by having the second workshop run face-to-face.

 “*Having you guys on site really got others on board, because they could see that you were really interested in trying to help*” (Interview, site coordinator, H10).

 Relationship building was also an important success factor. It enabled the external facilitator (VM) to develop an understanding of local practices, team dynamics, and the knowledge levels, which informed the tailored facilitation approach used during the educational outreach workshops and subsequent support period.

 All post-workshop survey respondents agreed that the external facilitator was knowledgeable and professional. This positive feedback was echoed by interview participants who described the external facilitator (VM) as *credible*, *approachable*, and *flexible*, and as playing a key role in ‘enabling’ and guiding teams through the action-planning process. The facilitator was seen as instrumental in this process, promoting teamwork and self-reflection to ensure locally relevant strategies were developed.

 “[The external facilitator]* had to slow us down a number of times … we would jump ahead to different areas without really teasing out some of the background behind it … So I think that was really helpful to have input and guiding us in that process” *(Interview, site coordinator, H2).

 Overall, the phone/email contacts from the external facilitator during the support period were generally described as beneficial, with the interactions often portrayed as “*check-ins*” and “*reminders or prompts”* for the site coordinator to follow-up with team members on the status of each strategy.

*“When someone externally would check in, it was a good reminder that we have to keep going. It was quite motivating really” *(Interview, site coordinator, H7).

 These contacts were reported as important in maintaining momentum with the local implementation efforts at each hospital, ensuring endeavours were focused on achievable outcomes, and that strategies were adapted when additional challenges arose.

 “[I could say] ‘*I’m having trouble with this,’ and *[the external facilitator] *would say, ‘well other sites have done a mini door-to-needle time audit on their prolonged thrombolysis cases, so maybe that is something you can look at.’ She didn’t say ‘You must do this, this and this,’ but there were some good suggestions about how we could go about things*” (Interview, site coordinator, H10).

 Although the number of contacts during the two-month support period was not pre-specified within the STELAR program, following delivery at the initial hospitals (step 1) the aim was for the external facilitator to maintain regular (eg, fortnightly) and proactive contact with hospital staff. Data from the support activity log indicated variation in these contacts (median 2, range 0-6) influenced by the part-time employment and occasional absences of both site coordinators and the external facilitator, as well as by local needs and staff expectations. Consequently, a “one size fits all” approach was not necessarily beneficial.

 “*Some sites didn’t want regular contact, like *[Hospital 7]*, they took ownership of their action plan and they went off and did it. I tried to initiate meetings with them but they sort of didn’t need them or didn’t want them. And I think that all that needs to be taken into account” *(Interview, External facilitator).

####  Focused Resources and Education

 At the end of the second workshop, 96% (n = 48) of respondents who completed the post-workshop satisfaction survey felt the structure of STELAR program was effective for reaching consensus on strategies to improve care. Overall, 90% were confident that the action plans could be implemented locally to change practice at their hospital. However, from free-text survey responses it was suggested that providing exemplar examples of what other hospitals had done—including the processes they followed and outcomes achieved—would have been useful. Similarly, during the interviews, site coordinators suggested that providing an explicit step-by-step implementation plan, that included examples of tools and documents (eg, discharge care plans), would have been of benefit.

 “*It may have been a misperception of me, but I was kind of expecting to see more examples of what other sites had done to help improve, and if they had similar barriers to us, this is what they used, or what they have done to address their gaps*” (Interview, site coordinator, H10).

 As part of the intervention, no specific training was provided to the site coordinators and change champions about the role or process of change management. To assist in implementing change, the potential benefit of further education related to the practicalities of how to engage the right people, and guidance related to implementing change management within STELAR was raised by two site coordinators during the interviews.

 Several site coordinators also reported that provision of a formal summary or final report post STELAR would have assisted to solidify the benefits of being involved in performance improvement initiatives for staff who were more peripherally involved and for hospital executives. The potential for a subsequent follow-up at 6-10 months down the track was suggested to sustain or continue to motivate the local team efforts and look to further embed changes in practice.

####  Role of the Local Change Champions (Internal Clinical Facilitator)

 In designing the STELAR program, it was anticipated that nominated change champions would lead local implementation efforts related to the respective quality indicator. However, action plan data showed that site coordinators took on the change champion role for over three quarters of the prioritized indicators chosen, regardless of hospital size or the number of staff involved with stroke care. This reportedly placed additional strain on site coordinator, who already faced limited capacity due to fractional appointments, existing workloads, and lack of flexibility within their existing roles, and was therefore perceived as a barrier to implementation of strategies locally. Implementation activities were further disrupted when staff in these positions had unexpected absences or resigned. Alternatively, in instances where site coordinators adopted a facilitating and enabling approach, supporting other staff as change champions and including them in the follow-up support contacts with the external facilitator, broader perceived benefits were reported. These included increased staff autonomy, shared accountability and responsibility, and enhanced capacity building in change management skills among the wider team.

 “*So, on reflection, maybe I didn’t do such a great job … maybe I should have stepped back … and I should have just been the facilitator for the workshop and then left the clinical champions to be the ones to liaise with *[the external facilitator] *and to have that accountability, rather than it coming back to me. If I did it again I would do it differently … If you show them how to do it and how they can be key clinicians in improving clinical care, then they can take responsibility and they are accountable for it*” (Interview, site coordinator, H5).

###  Innovation/Evidence

####  Data Driven Intervention Using Existing Performance Data 

 Interview participants reported that the components of the STELAR program were “*easy to follow*” and “*translatable*” to future quality improvement initiatives. Similarly, post-workshop survey data, and interviews highlighted that a strength of the program was that each hospital’s prioritized indicators were both data driven and self-selected, with strategies tailored to the local context, so were more likely to be successfully implemented.

 “[it was important to review]*… what we wanted to change, why things needed to change, talking through the issues and get to the bottom of the issues*” (Survey, nurse, H3).

 Additionally, using existing performance data collection via the AuSCR minimized the time and resource requirements for additional data collection and provided hospital-specific feedback related to recent care practices.

 “*It was good that we already have the AuSCR data, so there was no extra data collection that had to be undertaken, it was stuff that we were already doing”* (Interview, site coordinator, H8).

 The evidence-based clinical guideline recommendations that AuSCR data are based on were familiar to, and trusted by, hospital clinicians involved. However, post-survey responses and interview participants reported some concerns regarding AuSCR data quality, including missing data, effects of delayed data entry, difficulties with capturing data directly from electronic medical records, and local decisions to collect only a minimum number of AuSCR variables. Interview participants reflected on how these ongoing local issues with data extraction and completeness of records within AuSCR may have affected adherence to clinical practice outcome data being used to assess performance.

 An unanticipated consequence of being involved in the STELAR program was a reported enhanced understanded of the definitions concerning AuSCR data collection. This resulted in improved accuracy of clinical documentation in medical records across numerous hospitals, such as recording when a patient was mobilized.

####  Characteristics of the Action Plan

 Characteristics of the action plan were seen to influence the local implementation efforts both positively and negatively. The plan was for teams to identify 2-3 indicators for improvement taking into account the AuSCR performance data. However, analysis of action plans revealed an average of five prioritized indicators (range 1-7) were chosen. Most commonly, these related to receiving thrombolysis (if ischemic stroke) (N = 6 hospitals), receiving thrombolysis within 60 minutes of arrival at hospital (N = 6), swallow screen/assessment prior to oral intake (N = 6), discharged to community with a care plan (N = 6).

 On reflection, site coordinators felt that “more” was not always “better” when it came to the number of prioritized indicators and trying to focus attention on too many strategies ultimately affected overall implementation efforts. In reality, hospitals not only used data to identify gaps in performance, indicator selection also reflected pragmatic considerations, including feasibility, perceived importance, local capacity for change, or potentially indicators they felt motivated or comfortable working on. As a result, one hospital’s prioritized indicators did not align with the area showing the largest performance gap. While this what was not what the research team anticipate, the indicators chosen were still viewed as aspects of care where improvement was achievable.

 “*I think we mostly sort of decided what we wanted to go with anyway, areas that weren’t maybe reflected in the *[AuSCR]* data as areas we do terribly wrong, but something that we thought we could definitely improve on*” (Interview, site coordinator, H7).

 Interview data from site coordinators at four hospitals, indicated that certain strategies identified during the workshops were not actually feasible to implement due to staffing constraints or timeframes exceeding the two-month duration of the STELAR program.

 “*We decided not to do that one *[focus on delivery of thrombolysis] *in the end. We sort of started it, we looked at it, and we went this is too big for this project, and we are not going to focus on this” *(Interview, site coordinator, H8).

 The complexity of the identified strategies was also an important factor in local implementation. Challenges arose when strategies involved multiple professional disciplines, across multiple departments (ie, the emergency department and stroke unit) or required numerous steps to achieve. Therefore, complex strategies were often overlooked for strategies that were more straightforward to implement.

 “*If it was something simple, like I could just go out and make sure that the speech pathologists phone number was on every phone list, that’s easy to do*” (Interview, site coordinator, H8).

 Interview participants noted the benefit of having strategies underpinned by system or process changes and reinforced via specific resources. For example, the introduction of a new protocol was more likely to lead to sustainable practice change as compared to change being dependent on an individual, such as relying on reminders during ward rounds.

 “*The education package that has been developed – it is something that will be easily transferred from one clinician to the next if they leave the organization. Someone else will be able to lead the training package that has been developed*” (Interview, site coordinator, H5).

###  Recipients

####  Team Involvement

 Both interview and post-workshop survey data highlighted that involving key stakeholders throughout the entire process, from identifying practice gaps to developing realistic action plans and implementation, was seen as crucial to achieving the desired outcome. In total, 144 clinicians, including nurses (38%), allied health clinicians (30%), medical staff (9%), managers (10%), stroke coordinators (8%), and students (4%) attended the workshops. Overall attendance at the workshops was enhanced by combining with stroke educational days, stroke management meetings, or including in regular staff education time. Staff rotations and limited time were common challenges to workshop attendance, whereas rostering support from management and locating workshops within certain hospital departments (eg, Emergency Department) facilitated staff attendance. Considering a broad variety of clinical indicators (for example medical focused such as thrombolysis provision, or nursing and allied health related including discharge planning, and mobilization) was perceived as a catalyst for encouraging a wide range of disciplines to attend the workshops. Interview participants and survey respondents referred to the benefits of the team discussing local performance data, sharing views, generating ideas, and learning from each other at the workshop.

 “*I think it’s made us all a bit more aware of what we have to do as a team … we probably work a bit better as a team, instead of just sort of focusing on your own little area. So not in the silo as much*” (Interview, site coordinator, H4).

 Nevertheless, a common challenge was attracting relevant representatives from the Emergency Department and medical teams to attend workshops. Attendance data showed that medical representation was absent from both educational outreach workshops at five hospitals (56%). At two hospitals, this reportedly influenced the selection of indicators, with prioritized indicators chosen that did not rely on medical input. Additionally, engaging key stakeholders who were “*core*” to changing practice in the both the action plan development and implementation was considered critical. The specific stakeholders involved varied depending on the focus of the prioritized indicator. For example, speech pathologists and nurses were considered essential for implementing strategies related to swallow screening and assessment, while Emergency Department staff were key for strategies related to thrombolysis provision. As one site coordinator reflected:

 “*I think we maybe missed a group of people that would actually end up being crucial in the implementation of the discharge care plan. That was the *[Associate Nurse Unit Manager] *group on the ward, or the nurse in charge … from a day to day perspective, having someone sign off on the care plan and making sure it was complete, and then actually giving a copy to the patients. That’s actually where it fell down*” (Interview, site coordinator, H7).

 Ensuring the right team composition, along with adequate breadth of staff involvement, was critical to developing relevant strategies. Workshop observations, however, indicated limited engagement at three hospitals, with only one or two staff participating in action plan development.

 Overall, local implementation relied on the motivation and engagement of the change champions and other key stakeholders. A critical success factor was having a dynamic change champion, who prioritized quality improvement among their other responsibilities.

 “*I think the commitment of the driver … some people were on top of it, and other people, it was like at the back of a pile of all the other things they had to do, and it didn’t get enough of a look at”* (Interview, site coordinator, H6).

 Engagement in local implementation was often influenced by existing relationships between the site coordinator and the broader team. However, broader participation in local implementation was negatively affected by high workload demands and staff rotation.

###  Context

####  Organizational Context

 Various contextual influences impacted on the ability to effect practice change during the STELAR trial. Important to the successful engagement reported at some trial sites was ensuring synergises with existing complementary quality improvement programs that were already being undertaken. For example, two regional hospitals were involved in the Pulsara^TM^ pilot project, that had a focus on improving pre-hospital (ambulance) and hospital team communication,^28^ and other regional sites were able to access the Victorian Stroke Telemedicine program, which aimed to increase access to thrombolysis.^29^ Additionally, having the prioritized indicators identified in STELAR aligned with the existing organizational and unit priorities was also incentive for engagement.

 “*The discharge plan is drafted and will be put in the new stroke … pathway that we also happened to be updating at the time. So that was good timing for that*” (Interview, site coordinator, H10).

 External factors outside of participants’ control were reported as challenges at some hospitals. Competing organizational demands, such as the introduction of a new electronic medical record system, hospital redevelopment, and redesigned intranet for staff education, were challenges to overcome. These factors directly impacted on data collection and implementation of strategies that relied on integration within these systems. At numerous hospitals, a lack of existing systems or structures to support ongoing learning and the process of change was also reported as challenges to wider implementation and practice change.

 The organization of local stroke services was a further contextual barrier primarily reported at smaller regional hospitals. At these hospitals, generalist teams managed stroke as part of a larger case load, and they did not all work on a dedicated stroke unit with specialized staff. This reportedly influenced workload, and commitment to the program. Past involvement in prior quality improvement initiatives was generally understood to be a positive for engagement. However, at two hospitals, change fatigue and burnout were noted related to the demands of participating in numerous quality improvement projects.

 “*I think there can be a sense of burnout after time, specifically to me, but I suppose it could be to the team as well. Because if you’re saying ‘here is another quality initiative in stroke,’ the buy-in can be to some degree limited, because you have engaged the same people in multiple endeavours. There is a risk that they say, ‘Look stroke has had a fair shake, let’s give it a break for a while’*” (Interview, site coordinator, H1).

####  Management and Leadership Support

 As mentioned, there was limited leadership in the form of medical buy-in at the workshops, which was compounded by challenges with engagement of medical teams to assist in delivering and implementing the strategies across more than half the hospitals. This was an issue in hospitals where there was no dedicated medical lead for stroke, but also in larger hospitals where the head of stroke reportedly supported the program, but this did not translate into wider medical collaboration.

 “*It still very much a nursing lead discussion on ward rounds. To improve this further will probably need medical buy-in or a protocol so that this becomes standard practice*” (Interview, site coordinator, H2).

 At an organizational level, lack of management support related to insufficient allocation of resources or time to enter AuSCR data where challenges. In contrast, it was perceived that working with other groups within the hospital was important to support sustainability. For example, site coordinators discussed the benefits of involving the hospital quality improvement team to embed the culture of continuous quality improvement into clinical practice.

####  Inter-organizational Collaborations

 As STELAR was a multi-centre trial, a sense of collaborative effort to improve stroke care across the state was reported as a positive. Involvement with the Regional Stroke Network of stroke coordinators was also reported to have a positive influence on inter-organizational collaboration and resource sharing. The potential to strengthen the collaboration and share lessons, experiences and solutions between hospitals was perceived as beneficial for future quality improvement initiatives and collaborations.

###  Successful Implementation

####  Implementation Versus Practice Change

 Despite the STELAR program’s inclusion of two-months of remote-support from an external facilitator, the evaluation’s stepped-wedge design meant that the final post-intervention assessment occurred 10-12 months later for the hospitals allocated to the first two steps. This was important considering interview participants reported that implementation of some strategies may not lead to an immediately identifiable change in practice. Many felt that more than two-months would be required to see quantifiable changes in practice, as measured by the AuSCR data. For instance, new patient discharge care plans/forms were developed at four hospitals. However, at two of these hospitals, immediate use was hindered by the necessity for internal hospital governance approval before they could be rolled out for routine use, while additional staff training was considered essential at other hospitals.

 “*I feel like we’re still in the implementation phase. I think if you’re sort of asking about have we seen any improvement and results, no, we wouldn’t have because things are still in place. So, to judge if it has made a difference in our key performance indicators, we are not going to see that for a longer period of time. I think it’s been successful in terms of implementing changes,… that’s happening now, but if you look at the data that’s getting put into AuSCR, we’re still not going to see any improvement in the next month because they are not actually all rolled out yet*” (Interview, site coordinator, H10).

 Four site coordinators also felt that being involved with the STELAR program improved their confidence to use AuSCR data to leverage change within the organization and facilitate discussions with the wider team related to improvement areas. Moreover, participation resulted in heightened awareness of stroke care standards and quality expectations among clinicians, managers and hospital executives within participating organizations. Another broader benefit of participation in the STELAR program was the renewed overall focus on stroke care. While not directly tied to the prioritized indicators in their action plans, several hospitals had initiated or were planning additional stroke-related initiatives, such as more structured stroke orientation for staff and simulation training. These efforts capitalized on the enthusiasm and interest sparked by their involvement in the STELAR program.

 “*We have run a mock ‘code stroke,’ and we got a lot of people there for that, and I’m hoping to run a few more of those … we now have a *[Medical Registrar] *working here until August and she is really keen on stroke so hopefully we might get a bit more medical buy in over the next few months and run a few more of those sessions with the medical staff and emergency department staff” *(Interview, site coordinator, H4).

 Although it was generally reported that planning was underway for ongoing improvement activities after the STELAR support period finished, seven site coordinators were unable to provide an updated summary of achievements at the time of their interview. Some respondents indicated that monitoring progress was more difficult without the external “nudging” of the project team.

 Overall, Table 3 presents a summary of the key success factors and challenges identified from all data sources, offering insights that may support adaptation for real-world implementation.

**Table 3:** Summary of Success Factors and Challenges Related to the STELAR Program Offering Insights for Real-World Adaptation

**Success Factors**	
• External facilitation – value in developing locally relevant action plans and strategies, and during implementation for support/nudging	
• Data-driven selection of indicators for improvement – use of trustworthy, routinely collected data (eg, registry data)	
• Hospitals self-select indicators to increase relevance and likelihood of implementation	
• Focus on smaller number of indicators for improvement at one time (eg, 2-3)	
• Engage discipline-specific staff tied to each indicator (eg, ED staff for thrombolysis delivery) and set realistic timelines to ensure feasible, effective implementation	
• Dynamic clinical champions are important to drive change, but sharing responsibility among numerous clinicians is important for capacity building and successful improvement efforts	
• Consider process and system-driven change which are more effective than strategies that rely on individuals	
• Revisit plans and strategies to change or adopt	
• Align with wider organizational priorities	
• Encourage inter-organizational collaborations to share lessons, experiences and solutions	
**Challenge Identified**	**Potential Adaptation**
• Variation in facilitation contacts	• Tailor approach based on local needs
• Limited staff knowledge in behaviour change and practices of implementation	• Additional training in these areas • Use of templates or guidance tools
• Complex strategies, with multi-department or multi-step strategies harder to implement	• Allocate sufficient time for engagement and coordination across all relevant departments • Consider smaller, multi-step, more manageable strategies
• Wider workload constraints influencing capacity and time for focused implementation	• Consider timing of quality improvement initiatives relative to other activities • Phased or staggering implementation to reduce burden
• Limited leadership support from management and the executive	• Early and on-going relationship building important • Consider varied engagement activities
• Wider contextual disruptions including organizational change (eg, redevelopment)	• Adapt strategies to fit evolving environment, including mitigation plans
• Insufficient time for changes to be embedded in practice	• Develop realistic expectation for change related to real-world implementation • Maintain flexibility in timelines
• Reduced momentum once the research support ends	• Integrate ongoing support within existing hospital quality structures/broader networks • Monitoring or reporting mechanisms to sustain focus/accountability

Abbreviations: STELAR, Shared Team Efforts Leading to Adherence Results; ED, emergency department.

## Discussion

 Results of the STELAR program demonstrated an overall 17% (range 3% to 30%) improvement in the composite outcome.^16^ The findings presented in this paper highlight the critical factors for successful practice change. Specifically, the significance of support from implementation experts, and having evidence and choice underpinning the selection of the prioritized indicators was emphasized. Additionally, ensuring key stakeholders were involved in the facilitated action plan development to identify realistic strategies, and building capacity and sharing the responsibility for driving the implementation efforts locally were key. Addressing ongoing challenges including workload limitations, maximising medical buy-in and management support for such initiatives, and providing further education related to behaviour change and implementation may optimize the quality improvement intervention even further.

 The importance of the STELAR program using national clinical registry data (AuSCR) was highlighted. While ensuring the quality and completeness of these data was regarded as essential, specific care gaps could be readily identified. In practice, hospitals also considered feasibility, perceived importance, motivation, and local capacity when selecting indicators, meaning that prioritized indicators did not always correspond to the areas with the largest performance gaps. Nevertheless, this approach resulted in locally relevant indicators that were actionable and meaningful to each team. Given that locally initiated practice changes are more likely to be adopted,^30^ this may explain the greater success observed in changing practice compared to interventions targeting prespecified areas regardless of baseline performance or local preference.^31-33^ However, one identified challenge was that some teams were overly ambitious in the number of indicators prioritized. Similar to prior research,^34^ site coordinators reported that trying to focus on change for numerous clinical indicators at once was challenging, primarily due to the workload and wider staff engagement needed. Results from previous studies have suggested that targeting well-defined strategies for fewer indicators (eg, 2-3) is potentially more achieveable.^15^ Additionally, the complexity of strategies required to change practice was a consideration. Site coordinators described that simple changes were more easily achieved than strategies that required more complex change or collaboration between disciplines or across departments, which is consistent with prior research.^35^ This has particularly been reported as a challenge for delivery of thrombolysis,^36,37^ which involves, multiple departments, processes and medical buy-in. No improvement in the proportion of patients treated with thrombolysis within 60 minutes was seen in our main trial.^16^ In addition to the complexity involved, this finding may also be related to the small sample size for this indicator.^16^ Further, certain strategies within the action plans were not deemed feasible or realistic to implement without significant adaption, or within the trial’s timeframe. Overall, it was important to be realistic when developing action plans and revisit the plans during the implementation process to change or adapt these, especially when time is constrained to a set period.

 The external facilitator was considered key in assisting teams to develop action plans and processes to support local consensus activities. Contacts by the external facilitator during the two-month support period appeared particularly important to keep hospital staff focused, which could be considered a “nudge” strategy to influence behaviour and decision-making.^38^ Although the external facilitation component was relatively “light touch” compared with other interventions involving facilitation,^39,40^ the delivery and regularity of these contacts varied. This may be expected considering the part-time employment of many site coordinators, and the tailored nature of the intervention. However, similar challenges in delivering a “dose” for remote contacts has also been reported in prior research involving external facilitation.^41^ Although external facilitation delivered at higher intensities, and for longer, has resulted in improved effectiveness of evidence translation in primary care settings,^42^ a real-world approach is probably necessary. Potentially more advantageous in future work is having flexibility related to the remote-support. For example, clearly identifying the local needs and expectations related to these “contacts” so interactions are tailored to the requirements of the recipients within each context.

 Successful practice change requires both time and commitment.^43^ At some hospitals in the STELAR trial, a challenge to successful practice change was that the site coordinator was nominated as change champion for most indicators. Although these site coordinators displayed characteristics of “novice internal facilitators,” or individuals recognized for their strong interpersonal and interactive skills,^44^ they did not fully adopt the core function of facilitation, which centres on enabling others to take ownership and drive change.^45^ Site coordinators who took on the clinical champion role often experienced increased workload and strain. In contrast, some site coordinators used more of a facilitation-based approach, supporting other team members to act as change champions. This fostered autonomy, shared accountability, ownership and capacity building within the organization, which are all important aspects in sustainable practice change.^46^ Future quality improvement initiatives should engage a broader group of “change champions,” each responsible for specific indicators, and provide targeted training in facilitation, team building, and behaviour change skills.^44,47^

 In the present study, limited clinical leadership support, particularly from medical staff was perceived to have hampered the effectiveness of local implementation efforts at some sites for certain indicators. This aligns with recent research on implementing evidence-based protocols in emergency departments.^48^ Consideration to novel strategies to engage medical professionals in improvement initiatives is essential in future work to enhance access to evidence-based care. Providing more explicit implementation plans and wider sharing of documents and successful information-related processes and strategies from previous hospitals was a further step participants felt would enhance the program, which align with principles of Quality Improvement Collaboratives.^49^

 The concept of successful implementation versus practice change is also important to consider, with respect to the time-period required for change to occur.^50^ Although the support period was only two months, the stepped-wedge design meant that the post-intervention period across hospitals ranged from 2-12 months.^16^ At the end of the support period, site coordinators generally considered that many identified strategies were underway or had been successfully implemented. However, it was commonly perceived that a longer time may be required for the impact of all the implementation efforts to be reflected in the AuSCR performance data. Interestingly, adherence to some indicators not identified in the action plans also significantly improved in the post-intervention period.^16^ The reasons for this could, in part, be explained by the broader benefits of having a spotlight on improving overall stroke care as part of the STELAR program.

 Creating sustainable change beyond the study period is an important consideration with quality improvement initiatives,^51^ and is often challenging.^31^ As the STELAR program was data driven, being aligned with the AuSCR, continued routine monitoring of performance by the site coordinators was possible. However, the interview data suggested that the impetus to continue implementing the identified strategies waned after the support period ended. Responding to feedback from site coordinators, individual hospital reports were provided by the research team when the trial analysis was completed in 2019. These hospital reports provided program outcomes and a summary of changes to care processes identified in individual hospital action plans. In future, more contact or ‘nudging’ by the AuSCR support team, or further engaging each hospital’s quality improvement team, may encourage a more supporting culture around continuous quality improvement.^22^

 Although process evaluation data were collected in 2017/2018, now several years ago and prior to the COVID-19 pandemic, we believe the identified success factors and challenges remain relevant and applicable to current healthcare delivery contexts. A strength of this evaluation was that it was planned *a priori,* using a range of quantitative and qualitative data sources, and conducted by a team not directly involved in the delivery of the STELAR program. Considering the intervention delivery at the cluster or organizational level, and the local implementation, with use of the i-PARIHS framework, added depth to the analysis and interpretation of results lending to a more informed understanding of factors that influenced change in practice. Using two complementary but distinct frameworks provided both structure for collecting data and theoretical depth to interpret the data, enhancing the rigor of the evaluation.^52^ Data were collected at multiple time points across the trial, allowing insights from different stages to be incorporated into the analysis. Most findings identified through the triangulation process were convergent or complementary, reflecting consistent or different but compatible insights across data sources, rather than divergent. Nevertheless, the multiple triangulation approach offered a more complete exploration and understanding of factors that influenced quality improvement strategy adoption and subsequent practice change.^53^

 Author TP who led this analysis explicitly considered a range of assumptions and perspectives to minimize the potential of social desirability bias. As interviews were conducted at the completion of the STELAR trial, it was possible that participants may not have recalled all specific details of the delivery and implementation process. All site coordinators, who were key in local implementation efforts, and the external facilitator, participated in the interviews, thereby providing sufficient richness of data and information power.^54^ However, the potential benefit of gaining perspectives and further insights from other change champions related to their experiences is acknowledged, particularly related to the local implementation efforts.

 While this trial included hospitals in both urban and nonurban locations, with a variety of generalist and stroke specialist centres,^16^ our results are not directly generalizable to other countries, with different healthcare systems, models of stroke care and resourcing. However, variation in practice is an ongoing problem, and is not unique to the condition of stroke. Therefore, the structure of the STELAR program, particularly the synergistic benefit in using robust clinical registry data alongside external facilitation to help local staff interpret their data and plan for local improvement, can be adapted for use in developing countries or disease groups where quality of care in hospitals is measured. Rather than to test or prescribe specific solutions, the identified success factors and challenges provide important insights that can be used to tailor context-specific adaptations and implementation strategies.

 The evidence from this evaluation is also crucial for informing other quality improvement programs. It highlights the importance implementing and optimizing feedback strategies within national clinical quality registries (audit and feedback strategy). Emphasizing more regular communication or ‘nudging’ with local hospitals about quality improvement initiatives, along with evidence-based quality improvement programs to support effective use of registry data.

## Conclusion

 The multifaceted strengths of STELAR underpinned the improvements in stroke care quality, despite the challenges imposed by the limited implementation period as part of the research design. Important to broader quality improvement initiatives, success depends on providing adequate time to implement and review strategies, drawing on external knowledge translation expertise, developing internal staff capacity, and securing active support from medical and executive leadership. Reflecting on the critical success factors and ongoing challenges reported in this article is relevant to optimizing other audit and feedback programs when implementing similar quality improvement interventions.

## Acknowledgements

 The authors acknowledge staff from the participating hospitals, and clinicians who collect data for the AuSCR from the Victorian hospitals that were involved in the STELAR trial.

## Disclosure of artificial intelligence (AI) use

 Not applicable.

## Data availability statement

 Data not available as participants were informed and consented to only non-identifiable excerpts of their data being used in publications, they did not consent to data sharing.

## Ethical issues

 The STELAR trial was approved by the Melbourne Health Human Research Ethics Committee (HREC/16/MH/273), with local approval obtained from each participating hospital. No additional funding was provided for hospitals to undertake this study.

## Conflicts of interest

 Dominique A. Cadilhac reports being the Data Custodian for the AuSCR. Sandy Middleton and Monique F. Kilkenny are members of the AuSCR Steering and Management Committee, respectively. Violet Marion and Julie Morrison are employed as coordinators for AuSCR with regular contact with hospitals. Other authors declare that they have no conflicts of interest.

## Supplementary files


Supplementary file 1. Additional Details of STELAR Methods.

Supplementary file 2. COREQ (COnsolidated criteria for REporting Qualitative research) Checklist.

Supplementary file 3. Data Collection Documentation.

Supplementary file 4. Summary of the Clinician Site Coordinator Characteristics.

Supplementary file 5. Triangulation Matrix.
