# Supplementary material for: Process Evaluation of an Effective Multifaceted Quality Improvement Intervention to Improve Acute Stroke Care: Unpacking the Success Factors and Challenges
Source: Int J Health Policy Manag. 2026 Mar 10;15:9013. doi: 10.34172/ijhpm.9013 (PMC13145233; doi:10.34172/ijhpm.9013)
Supplement: Supplementary file 1 — Additional Details of STELAR Methods. [file ijhpm-15-9013-s001.pdf]

Article title: Process Evaluation of an Effective Multifaceted Quality Improvement Intervention to Improve Acute Stroke Care: Unpacking the Success Factors and Challenges

Journal name: International Journal of Health Policy and Management (IJHPM)

Authors' information: Tara Purvis[1]*, Elizabeth Lynch[2], Violet Marion[3], Julie Morrison[3], Monique F. Kilkenny[1,3], Sandy Middleton[4,5], Dominique A. Cadilhac[1,3]

[1]Department of Medicine, School of Clinical Sciences at Monash Health, Monash University, Clayton, VIC, Australia.

[2]College of Nursing and Health Sciences, Flinders University, Adelaide, SA, Australia.

[3]Stroke and Critical Care Research, The Florey Institute of Neuroscience and Mental Health, University of Melbourne, Heidelberg, VIC, Australia.

[4]Nursing Research Institute, St Vincent's Health Network Sydney, St Vincent's Hospital Melbourne and Australian Catholic University, Sydney, NSW, Australia.

[5]School of Nursing Midwifery and Paramedicine, Australian Catholic University, Sydney, NSW, Australia.

***Correspondence to:** Tara Purvis; Email: tara.purvis@monash.edu

Citation: Purvis T, Lynch E, Marion V, et al. Process evaluation of an effective multifaceted quality improvement intervention to improve acute stroke care: unpacking the success factors and challenges. Int J Health Policy Manag. 2026;15:9013. doi:10.34172/ijhpm.9013

Supplementary file 1. Additional Details of STELAR Methods

- **Methods of the STELAR program**
- **Table S1: Delivery and modifications to the delivery of the STELAR program to hospitals (clusters)**

Methods of the STELAR program

The STELAR program was delivered to nine participating hospitals (urban area n=2, nonurban area n=7) sequentially in three steps, with an iterative approach that permitted lessons from the intervention delivery at the first block of hospitals to refine the intervention for subsequent hospitals (Supplementary file 2 Table S1). Each participating hospital nominated a clinician site coordinator who was responsible for inviting all multidisciplinary staff involved with stroke care or improvement initiatives within the hospital to be part of the STELAR program. Hospital staff were asked to complete a pre-workshop survey to identify perceived areas of practice gaps, and barriers that might be impacting performance.

The first stage of the program intervention included an educational outreach workshop delivered via videoconference by the external facilitators; lead researcher DAC (AuSCR data custodian, and expert in health service evaluation) and/or VM (Project coordinator, with clinical background in nursing and previous experience in facilitation and knowledge translation projects). Hospital pre-workshop survey results regarding perceived areas of practice gaps and local performance data related to the 10 AuSCR clinical quality indicators, representing evidence-based acute stroke treatments or therapies, were summarized and discussed (Supplementary file 2 Table S1). Local AuSCR data were benchmarked against the aggregated national data, and to that of peer hospitals of a similar size and region (e.g. urban/nonurban).

Facilitated discussions by VM assisted hospital staff to collaboratively identify priority clinical quality indicators (herein referred to as indicators) for focussed improvement activities as part of STELAR, with discussions of the potential modifiable barriers to care delivery related to these indicators. VM also facilitated the second educational outreach workshop which was held face-to-face at the hospitals within a month of the first. The second workshop included education about the evidence supporting the identified indicators, followed by development of an action plan. This included detailed documentation of the agreed evidence-based strategies to improve delivery of the prioritized indicators using the pre-specified action plan template (Supplementary file 7 Figure S1). Individualized, tailored, strategies were encouraged within the broader context of the Effective Practice and Organisation of Care (EPOC) Taxonomy (2015 https://epoc.cochrane/epoc-taxonomy [accessed July 2024]). As part of the strategy development, staff nominated a local 'change champion' to take responsibility for ensuring the implementation of the action plan and the agreed strategies for each indicator. This change champion was any member of the multidisciplinary team who had responsibility for the provision of the clinical indicator. Following the second workshop, VM provided two-months of ongoing support to the change champions via telephone or email and recorded all contacts in a support activity log.

For more detailed information about the methods please see the primary outcome paper by Cadilhac et al (Joint Commission Journal on Quality and Patient Safety. 2022;48(12):653-664).

Table S1: Delivery and modifications to the delivery of the STELAR program to hospitals (clusters)

**Clinical Indicators collected in the Australian Stroke Clinical Registry (AuSCR)**		
Treated in a stroke unit	Swallow screen/assessment prior to oral intake	Discharged on antihypertensive medication
Received thrombolysis, if ischemic stroke	Aspirin given as hyperacute therapy (ischemic)	Discharged on antithrombotic medication (ischemic)
Received thrombolysis in 60 min of arrival (ischemic)	Discharged to community with a care plan	Discharged on lipid lowering medication (ischemic)
Mobilized on day of admission or day following		
**Delivery of STELAR intervention – 'as planned'**	**Modifications to delivery**	**'What was delivered' and 'to whom'**
Pre-workshop survey - Determine perceived areas of practice gaps, and related barriers to providing best practice care		
• Site coordinators emailed survey 2-3 weeks prior to W1<br>• Input from multiple staff at each hospital encouraged<br>• Completed surveys returned to project team prior to W1 to form basis of discussion at W1	• After step 1 hospitals, electronic option provided to complete pre-survey<br>• Modification of language used for questions relating to barrier identification due to poor response	• Pre-survey completed by 8/9 hospitals (N=5 within 1 day of W1)<br>• Collaborative/multiple responses from 7 hospitals<br>• Only 1 pre-survey had all sections related to barrier identification complete
Facilitated educational outreach workshops		
Workshop 1 – Data feedback (one at each hospital, teleconference, 60 min)		
• Perceived barriers from pre-survey discussed<br>• Presentation of local clinical performance data (AuSCR) compared to peer & national benchmarks (VM)<br>• Hospital staff collaboratively prioritize 2-3 clinical indicators for improvement based on AuSCR data presented (VM/DAC)<br>• Staff discuss potential modifiable barriers to care delivery for prioritized indicators (VM facilitates)<br>• Staff to complete barrier identification activity for prioritized indicators independently and return to project team prior to W2 – used to tailor focus in W2	• No major changes from 'as planned'	• 102 staff attended (median 12/hospital)<br>• No medical attendance at 5 hospitals<br>• Slides emailed to site coordinator after workshop
Workshop 2 – Tailored education and facilitated action planning (one at each hospital, face to face, 2 hours, 2-3 weeks after W1)		
• Education provided by 'local opinion leader' on prioritized indicators for improvement (slides developed by VM)<br>• Education around theory of quality improvement & behaviour change (VM)<br>• Staff work with VM to develop action plan to outline locally relevant strategies to address modifiable barriers to care delivery for prioritized indicators	• After step 1 hospitals:<br>- conscious decision by VM to allocate less time for review of AuSCR data, education, and theory related to behaviour change, to allow more time for barrier identification and facilitated action planning<br>- Electronic option for satisfaction survey completion	• At 3 hospitals, action plan completed by 1-2 key staff during W2 (others had to leave W2 early)<br>• Average of 5 indicators were identified for improvement at each hospital (range 1-7)

Workshop 2 – Tailored education and facilitated action planning (one at each hospital, face to face, 2 hours, 2-3 weeks after W1) – cont'd		
• Staff nominate 'change champions'<br>• Hardcopy satisfaction surveys distributed	• W2 occurred 85 days after W1 (one hospital); an additional meeting with local staff was held to further unpack AuSCR data prior to W2	• Nominated change champion was the site coordinator for 78% of all indicators
Remote support period - to support implementation of local strategies related to action plans (2 months from finalization of action plan by external facilitator)		
• Direct contact by VM with change champion/site coordinator via email or phone coordinators to assist in implementing local strategies related to action plans<br>• Contacts recorded by VM in support activity log	• After step 1 hospitals, aiming for regular proactive fortnightly contacts with sites rather than ad hoc contact	• Support period began between 1-33 days after W2 (action plan finalised by VM)<br>• Direct phone/email contact provided to staff at 8/9 hospitals (median 2 contacts; range 0-6)<br>• 5 hospitals had post intervention interview with VM<br>• Contacts for support made with only site coordinator at 5 hospitals

Step 1 consisted of first 2 hospitals; W1 – first workshop; W2-second workshop; AuSCR- Australian Stroke Clinical Registry; DAC- project staff, VM – project staff, both considered as external facilitators
