# Supplementary material for: Process Evaluation of an Effective Multifaceted Quality Improvement Intervention to Improve Acute Stroke Care: Unpacking the Success Factors and Challenges
Source: Int J Health Policy Manag. 2026 Mar 10;15:9013. doi: 10.34172/ijhpm.9013 (PMC13145233; doi:10.34172/ijhpm.9013)
Supplement: Supplementary file 2 — COREQ (COnsolidated criteria for REporting Qualitative research) Checklist. [file ijhpm-15-9013-s002.pdf]

Article title: Process Evaluation of an Effective Multifaceted Quality Improvement Intervention to Improve Acute Stroke Care: Unpacking the Success Factors and Challenges

Journal name: International Journal of Health Policy and Management (IJHPM)

Authors' information: Tara Purvis[1]*, Elizabeth Lynch[2], Violet Marion[3], Julie Morrison[3], Monique F. Kilkenny[1,3], Sandy Middleton[4,5], Dominique A. Cadilhac[1,3]

[1]Department of Medicine, School of Clinical Sciences at Monash Health, Monash University, Clayton, VIC, Australia.

[2]College of Nursing and Health Sciences, Flinders University, Adelaide, SA, Australia.

[3]Stroke and Critical Care Research, The Florey Institute of Neuroscience and Mental Health, University of Melbourne, Heidelberg, VIC, Australia.

[4]Nursing Research Institute, St Vincent's Health Network Sydney, St Vincent's Hospital Melbourne and Australian Catholic University, Sydney, NSW, Australia.

[5]School of Nursing Midwifery and Paramedicine, Australian Catholic University, Sydney, NSW, Australia.

***Correspondence to:** Tara Purvis; Email: tara.purvis@monash.edu

Citation: Purvis T, Lynch E, Marion V, et al. Process evaluation of an effective multifaceted quality improvement intervention to improve acute stroke care: unpacking the success factors and challenges. Int J Health Policy Manag. 2026;15:9013. doi:10.34172/ijhpm.9013

Supplementary file 2. COREQ (COnsolidated Criteria for REporting Qualitative Research) Checklist

Table S2

Developed from: Tong A et al (2007, International Journal for Quality in Health Care; 19(6):249-257)

No.	Item	Guide questions/ description	Page
Domain 1: research team and reflexivity			
Personal Characteristics			
1	Interviewer/facilitator	Which author/s conducted the interview or focus group?	7
2	Credentials	What were the researchers credentials? *E.g. PhD, MD*	7
3	Occupation	What was their occupation at the time of the study	7
4	Gender	Was the researcher male or female?	7
5	Experience and training	What experience or training did the researcher have	7
Relationship with participants			
6	Relationship established	Was a relationship established prior to study commencement?	7,8
7	Participant knowledge of the interviewer	What did the participants know about the researcher? *E.g. personal goals, reasons for doing the research*	7,8
8	Interviewer characteristics	What characteristics were reported about the interviewer/facilitator? *E.g Bias, assumptions, reasons and interests in the research topic*	7,23
Domain 2: study design			
Theoretical framework			
9	Methodological orientation and theory	What methodological orientation was stated to underpin the study? *E.g. grounded theory, discourse analysis, ethnography, phenomenology, content analysis*	-
Participant selection			

10	Sampling	How were participants selected? *E.g. purposive, convenience, consecutive, snowball*	7
11	Methods of approach	How were participants approached? *E.g. face-to-face, telephone, mail, email*	7
12	Sample size	How many participants were in the study?	7,8
13	Non-participation	How many people refused to participate or dropped out? Reason?	7,8
Setting			
14	Setting of data collection	Where was the data collected? *E.g. home, clinic, workplace*	7
15	Presence of non-participants	Was anyone else present besides the participants and researchers?	7
16	Description of sample	What are the important characteristics of the sample? *E.g. demographic data, date*	Supplementary file 5 Table S4
Data collection			
17	Interview guide	Were questions, prompts, guides provided to the authors? Was it pilot tested?	7, Supplementary file 9 Figure S3
18	Repeat interviews	Were repeat interviews carried out? If yes, how many?	No
19	Audio/visual recording	Did the research use audio or visual recording to collect the data?	7
20	Field notes	Were field notes made during and/or after the interview of focus group	7
21	Duration	What was the duration of the interviews or focus group?	7
22	Data saturation	Was data saturation discussed?	23
23	Transcripts returned	Were transcripts returned to participants for comment and/or correction?	7
Domain 3: analysis and findings			
Data analysis			
24	Number of data coders	How may coders coded the data?	7,8
25	Description of the coding tree	Did authors provide a description of the coding tree?	7,8
26	Derivation of themes	Were themes identified in advance or derived from the data?	7,8
27	Software	What software, if applicable, was used to manage the data?	8
28	Participant checking	Did participants provide feedback on the findings?	No
Reporting			
29	Quotations presented	Were participant quotations presented to illustrate the themes/findings? Was each quotation identified? *E.g. participant number*	Throughout results
30	Data and findings consistent	Was there consistency between the data presented and the findings?	Throughout results
31	Clarity of major themes	Were major themes clearly presented in the findings?	Throughout results
32	Clarity of minor themes	Is there a description of diverse cases or discussion of minor themes?	Throughout results
