# Supplementary material for: Process Evaluation of an Effective Multifaceted Quality Improvement Intervention to Improve Acute Stroke Care: Unpacking the Success Factors and Challenges
Source: Int J Health Policy Manag. 2026 Mar 10;15:9013. doi: 10.34172/ijhpm.9013 (PMC13145233; doi:10.34172/ijhpm.9013)
Supplement: Supplementary file 3 — Data Collection Documentation. [file ijhpm-15-9013-s003.pdf]

Article title: Process Evaluation of an Effective Multifaceted Quality Improvement Intervention to Improve Acute Stroke Care: Unpacking the Success Factors and Challenges

Journal name: International Journal of Health Policy and Management (IJHPM)

Authors' information: Tara Purvis[1]*, Elizabeth Lynch[2], Violet Marion[3], Julie Morrison[3], Monique F. Kilkenny[1,3], Sandy Middleton[4,5], Dominique A. Cadilhac[1,3]

[1]Department of Medicine, School of Clinical Sciences at Monash Health, Monash University, Clayton, VIC, Australia.

[2]College of Nursing and Health Sciences, Flinders University, Adelaide, SA, Australia.

[3]Stroke and Critical Care Research, The Florey Institute of Neuroscience and Mental Health, University of Melbourne, Heidelberg, VIC, Australia.

[4]Nursing Research Institute, St Vincent's Health Network Sydney, St Vincent's Hospital Melbourne and Australian Catholic University, Sydney, NSW, Australia.

[5]School of Nursing Midwifery and Paramedicine, Australian Catholic University, Sydney, NSW, Australia.

***Correspondence to:** Tara Purvis; Email: tara.purvis@monash.edu

Citation: Purvis T, Lynch E, Marion V, et al. Process evaluation of an effective multifaceted quality improvement intervention to improve acute stroke care: unpacking the success factors and challenges. Int J Health Policy Manag. 2026;15:9013. doi:10.34172/ijhpm.9013

Supplementary file 3. Data Collection Documentation.

- **Table S3: Information included in the Support Activity log**
- **Figure S3a: Pre-specified action plan template**
- **Figure S3b: Participant post-workshop satisfaction survey**
- **Figure S3c: Facilitator semi-structured interview guide**

Table S3: Information included in the Support Activity log

Item	Description (as required)
Date of contact	
Who contact involved	e.g. Site coordinator, local champion, other team members
Mode of contact	Email, teleconference, telephone
Contact time (mins)	For telephone, teleconference
Indicator/s discussed	Based on prioritized indicators identified at each hospital
Type of strategy implemented*	Strategies implemented locally, for example audit and feedback, educational materials, education
Current progress	Summary of progress related to each strategy
Internal barrier/s	Internal barriers reported
External barrier/s	External barriers reported
New identified barriers	
Agreed action/s moving forward	
Planned next contact	
Comments	Other relevant details of interest e.g. progress

*Categorised by Effective Practice and Organization of Care Group (EPOC) (2015 https://epoc.cochrane/epoc-taxonomy [accessed July 2024])

Figure S3a: Pre-specified action plan template

Improvement Action Plan

(achieving short term and longer term goals)

Aims	Strategies	Who<br>How<br>When	Evaluation Measures

Figure S3b: Participant post-workshop satisfaction survey

Survey ID:________________ Hospital ID:_______________

Date survey completed: ____/____/20____

Profession:
- ☐ ADMINISTRATOR/EXECUTIVE
- ☐ DIETITIAN
- ☐ DOCTOR
- ☐ NURSE
- ☐ OCCUPATIONAL THERAPIST
- ☐ PHARMACY
- ☐ PHYSIOTHERAPIST
- ☐ SOCIAL WORKER
- ☐ SPEECH PATHOLOGIST
- ☐ OTHER____________________

Current role at this hospital:____

Number of months in current role:____

Age group (years): ☐ <24 ☐ 25-34 ☐ 35-44 ☐ 45-54 ☐ 55-64 ☐ 65+

Gender: ☐ Male ☐ Female

Employment status: ☐ Full Time ☐ Part Time ☐ Casual

Department / Unit:____

Were you involved in the following steps in relation to the STELAR Program?

Identified the evidence practice gaps (using the AuSCR data reports) ☐ Yes ☐ No

Identified the barriers/enablers to change (completed pre-workshop survey) ☐ Yes ☐ No

Participated in:
STELAR workshop 1 ☐ Yes ☐ No
STELAR workshop 2 ☐ Yes ☐ No

Assisted with Action Plan now developed during Workshop 2? ☐ Yes ☐ No

---

Please indicate with a X the most applicable response option:

**STELAR Program**	**Strongly agree**	**Agree**	**Neutral**	**Disagree**	**Strongly disagree**
I understand the purpose of the STELAR program					
I learnt new information					
The program level was appropriate					
I feel more confident to identify and prioritise practice gaps					
The pre-workshop survey evaluation was useful and helped to streamline the action planning process					
There was the right amount of opportunity for participation in all components					
I would recommend the program to others					
The content was relevant to my work					

Please indicate with a X the most applicable response option (check n/a if you did not attend workshop 1):

**STELAR Workshop 1** n/a ☐	**Strongly agree**	**Agree**	**Neutral**	**Disagree**	**Strongly disagree**
I intend to use the knowledge and skills gained in this workshop to improve my practice					
The activities and tools used during the workshop were useful					
The structure of the workshop helped me stay engaged					
The structure of the workshop was effective for reaching consensus on strategies to improve care					
The time allocated to the workshop was just right					
The facilitator made me feel comfortable to participate in discussions					
The facilitator was knowledgeable and professional					
I enjoyed participating in the workshop					

Please indicate with a X the most applicable response option:

**STELAR Workshop 2**	**Strongly agree**	**Agree**	**Neutral**	**Disagree**	**Strongly disagree**
I intend to use the knowledge and skills gained in this workshop to improve my practice					
The activities and tools used during the workshop were useful					
The education component provided by the local opinion leader was beneficial					
The structure of the workshop helped me stay engaged					

STELAR Workshop 2	Strongly agree	Agree	Neutral	Disagree	Strongly disagree
The structure of the workshop was effective for reaching consensus on strategies to improve care					
The workshop was pivotal in developing action plans relevant to our hospital					
I feel confident we can implement the action plans developed					
Identifying clinical change champions was valuable and will assist in the implementation process					
The time allocated to the workshop was just right					
The facilitator made me feel comfortable to participate in discussions					
The facilitator was knowledgeable and professional					
I enjoyed participating in the workshop					

Please describe what you found to be the *most valuable aspect/s of the STELAR Program*?

____

____

____

Do you have any suggestions for *improving the STELAR Program*?

____

____

____

Do you have access in your hospital to the InformMe (Stroke Foundation) website? ☐ Yes ☐ No

Do you use the resources available from the InformMe (Stroke Foundation) website? ☐ Yes ☐ No

Would it be helpful to have STELAR action planning tools and information accessible from (please circle):

a) InformMe (Stroke Foundation) website

b) AuSCR website

c) Victorian Stroke Clinical Network website

d) None of the above

Figure S3c: Facilitator semi-structured interview guide

Demographic info

- Male/Female
- Age
- Hospital
- Professional background
- Experience at current hospital/overall
- Involvement in any quality improvement activities/program prior to STELAR. Please explain

Participation in STELAR

1.Were you involved in: i) Pre-survey, ii) Workshop 1, iii) Workshop 2

Overall view on barriers/enablers to providing evidence-based stroke care, and what role clinicians, resources, health system and policy have

2. Prior to being involved in STELAR, what did you consider as the key barriers and facilitators in delivering evidence-based stroke care in your hospital? Specifically, relate back to processes of care (PoC) in action plan

   i) What role do the individual clinicians have

   ii) Do resources play a part

   iii) What role does current policy have

   iv) Is there wider support

Overall view on STELAR

3. What were your thoughts on the components of the STELAR trial overall?

   (e.g. Pre-survey, Workshop 1, Workshop 2, Implementation, Support)

Specific prompts:

   i) How many processes of care (PoC) were identified in the action plan that was developed for your hospital? Was this appropriate, too many/didn't cover all areas? Why?

   ii) How many PoC were you involved with? What were you working on as part of these action plans?

   iii) Can you comment on the strategies outlined in the action plans? a) Were they specific enough, helpful b) What, if anything would have improvement these action plans /strategies developed

   iv) Support period

4. How did you perceive the role of the external facilitator in the intervention delivery/local implementation process? - particularly relating to their key roles/attributes, specifically in;

   i) Workshops

   ii) Action planning

   iii) Support period--- what was offered, often enough, for long enough

   iv) What, if anything, would you recommend be changed with the involvement/role of the external facilitator

5. Was the intervention period long enough to bring about a behaviour change that was translated into practice change? Please explain. If not why not…how long do you think would be required?

6. What were the most helpful aspects of the intervention?

Prompts: data, getting all staff involved- collaboration, strategies, opinion leaders, support (have you looked at AuSCR data since)

General impressions- understand how the trial integrated into everyday practice

7. Have you received any feedback from staff regarding hospital involvement in STELAR?

8. Regardless of any changes being made, do you feel staff involved in stroke care altered their usual practice as a result of just being involved in STELAR

9. What was the impact on your unit of being part of this trial? Were there any negative impacts on your unit associated with participating in the trial? Were there any benefits? Were there any benefits to you as a clinician in participating in this trial?

Suitability of trial design

10. What are your thoughts on the outcomes used in this study?

11. Do you feel you were successful in bringing about change in the processes identified in the action plans- if yes, which areas and why, if no, why not …..

12. Are you satisfied with your change or otherwise in AuSCR data?

i) If checked

ii) If not checked, why not?
iii) Any concerns with data quality

13. Could you please rate your 'progress in achieving significant quality improvement' using the Institute of Healthcare Improvement scale below

Score	Definition
1	No activity yet (non-starter)
2	Activities implemented, no improvement yet
3	Modest improvement
4	Significant improvement
5	Outstanding progress ('best practice')

14. On reflection, do you feel the STELAR intervention achieved its desired objectives? Why? Why not?

Translation into clinical practice & policy

15. What do you think are the main barriers/facilitators to implementing further changes?

16. What would be your ideal model to assist stroke teams to improve care? Is there anything more than what was offered in the intervention that you would like to see?

17. Do you think that changes will be sustainable moving forward?....Is the process translatable to other areas/processes?

18. Would you be interested in continuing to participate in a larger version of this trial? If no, why not?

Role as clinical champion

19. Can you describe how you perceived your role as clinical champion for STELAR?

Prompt: leadership, project management, linkage, etc

i) What were the most important aspects/attributes/elements of the role?

20. What factors did you find assisted you in your role as a clinical champion/internal facilitator?

a. Personally

i. Skills- how easy did you find the role (skills- competence)

ii. Knowledge-know what you should be doing (knowledge- procedural knowledge)

iii. Training (behavioural regulation)

iv. Observation of others (social influences)

b. Sites (team/staff, data, organisational)

i. Resources/physical factors (environmental context and resources)

ii. Knowledge

iii. Skills

c. Wider stakeholders (organisational, colleagues)

i. Existing networks/new networks

ii. Managers, other professional groups etc (social influences)

21. What factors did you find made your role as a clinical champion/internal facilitator difficult? Barriers to role

a. Personally

i. Capabilities/emotion

ii. Skills- how easy did you find the role (skills- competence)

iii. Knowledge-know what you should be doing (knowledge- procedural knowledge)

iv. Training/manuals (behavioural regulation)

v. Observation of others (social influences)

b. Sites

i. Resources/physical factors (environmental context and resources)

ii. Competing time constraints (environmental context and resources)

iii. Conflicts

c. Wider stakeholders/organisational

22. What feedback, if any, have you received (informal or formal)
23. If you had your time again with this project, would there be anything that you would change as part of your role as a clinical champion (perceived competence- beliefs about capabilities, nature of behaviours)
24. Is there anything further you would like to add that we have not talked about?
