# Supplementary material for: Process Evaluation of an Effective Multifaceted Quality Improvement Intervention to Improve Acute Stroke Care: Unpacking the Success Factors and Challenges
Source: Int J Health Policy Manag. 2026 Mar 10;15:9013. doi: 10.34172/ijhpm.9013 (PMC13145233; doi:10.34172/ijhpm.9013)
Supplement: Supplementary file 4 — Summary of the Clinician Site Coordinator Characteristics. [file ijhpm-15-9013-s004.pdf]

Article title: Process Evaluation of an Effective Multifaceted Quality Improvement Intervention to Improve Acute Stroke Care: Unpacking the Success Factors and Challenges

Journal name: International Journal of Health Policy and Management (IJHPM)

Authors' information: Tara Purvis[1]*, Elizabeth Lynch[2], Violet Marion[3], Julie Morrison[3], Monique F. Kilkenny[1,3], Sandy Middleton[4,5], Dominique A. Cadilhac[1,3]

[1]Department of Medicine, School of Clinical Sciences at Monash Health, Monash University, Clayton, VIC, Australia.

[2]College of Nursing and Health Sciences, Flinders University, Adelaide, SA, Australia.

[3]Stroke and Critical Care Research, The Florey Institute of Neuroscience and Mental Health, University of Melbourne, Heidelberg, VIC, Australia.

[4]Nursing Research Institute, St Vincent's Health Network Sydney, St Vincent's Hospital Melbourne and Australian Catholic University, Sydney, NSW, Australia.

[5]School of Nursing Midwifery and Paramedicine, Australian Catholic University, Sydney, NSW, Australia.

***Correspondence to:** Tara Purvis; Email: tara.purvis@monash.edu

Citation: Purvis T, Lynch E, Marion V, et al. Process evaluation of an effective multifaceted quality improvement intervention to improve acute stroke care: unpacking the success factors and challenges. Int J Health Policy Manag. 2026;15:9013. doi:10.34172/ijhpm.9013

Supplementary file 4. Summary of the Clinician Site Coordinator Characteristics

Table S4

**Characteristic**	**N=9**<br>**n (%)**
Female	7 (78%)
Age (median, Q1, Q3)	40 (30, 50)
Profession	
Nurse	7 (78)
Allied health*	2 (22)
Years in current role (median, Q1, Q3)	2 (1.25, 5)
Full time equivalent ≤ 0.5	7 (78%)
Prior experience in quality improvement	All had prior experience with collection of data for the AuSCR and the Stroke Foundation National Audit Program^, and local quality improvement initiatives. All experienced in their professional role, but varied level of experience with coordination activities

Q1-1st quartile; Q3- 3rd quartile; AuSCR- Australian Stroke Clinical Registry;*speech pathologist, physiotherapist; ^biennial national audit of stroke services
