# Supplementary material for: Process Evaluation of an Effective Multifaceted Quality Improvement Intervention to Improve Acute Stroke Care: Unpacking the Success Factors and Challenges
Source: Int J Health Policy Manag. 2026 Mar 10;15:9013. doi: 10.34172/ijhpm.9013 (PMC13145233; doi:10.34172/ijhpm.9013)
Supplement: Supplementary file 5 — Triangulation Matrix. [file ijhpm-15-9013-s005.pdf]

**Article title:** Process Evaluation of an Effective Multifaceted Quality Improvement Intervention to Improve Acute Stroke Care: Unpacking the Success Factors and Challenges

**Journal name:** International Journal of Health Policy and Management (IJHPM)

**Authors' information:** Tara Purvis[1]*, Elizabeth Lynch[2], Violet Marion[3], Julie Morrison[3], Monique F. Kilkenny[1,3], Sandy Middleton[4,5], Dominique A. Cadilhac[1,3]

[1]Department of Medicine, School of Clinical Sciences at Monash Health, Monash University, Clayton, VIC, Australia.

[2]College of Nursing and Health Sciences, Flinders University, Adelaide, SA, Australia.

[3]Stroke and Critical Care Research, The Florey Institute of Neuroscience and Mental Health, University of Melbourne, Heidelberg, VIC, Australia.

[4]Nursing Research Institute, St Vincent's Health Network Sydney, St Vincent's Hospital Melbourne and Australian Catholic University, Sydney, NSW, Australia.

[5]School of Nursing Midwifery and Paramedicine, Australian Catholic University, Sydney, NSW, Australia.

***Correspondence to:** Tara Purvis; Email: tara.purvis@monash.edu

**Citation:** Purvis T, Lynch E, Marion V, et al. Process evaluation of an effective multifaceted quality improvement intervention to improve acute stroke care: unpacking the success factors and challenges. Int J Health Policy Manag. 2026;15:9013. doi:10.34172/ijhpm.9013

**Supplementary file 5.** Triangulation Matrix

**Table S5: Triangulation matrix**

| Theme/sub-theme | Workshop observations and/or documentation* | Post-workshop survey | Support Activity Log | Interviews | Triangulation |
|---|---|---|---|---|---|
| Facilitation | | | | | |
| Benefit of involvement of external organisation | Fostered engagement – 144 staff attended workshops, strong multidisciplinary involvement | Bought team together – value in cross-disciplinary discussions | | Endorsed across all hospitals | Convergent |
| External facilitator role | Observed high engagement when facilitator led workshops | 100% rated facilitator "*knowledgeable*" and "*professional*" | Variation in number and type of contacts during support period | External facilitator described as motivating and guiding; benefit in tailored approach to support | Complimentary - Divergent – Benefits in different aspects of role during workshops and action planning + variation during support period |
| Internal facilitator role of site coordinator | Site coordinators nominated 'local champion' for most prioritized indicators | | Contacts made during support period were primarily with site coordinators only | Barriers to role identified (workload, limited capacity); benefits in enabling other team members for shared accountability/capacity building | Complementary |
| Implementation resources and education | | 96% respondents felt the structure of STELAR program was effective for reaching consensus on strategies to improve care<br><br>Free text – exemplar examples of practical strategies would be helpful | | Value in shared learning between hospitals; desire for additional behaviour-change education | Complimentary – Divergent – recognition in value of structure of program + desire for additional resources |
| Innovation | | | | | |
| Data-driven approach using routine registry data | Broad range of indicators important for multidisciplinary team involvement;<br>Not all prioritized indicators represented the largest performance gaps | Benchmarking performance against peer hospitals valued; benefit in self-selecting areas for improvement; concern about effect of delayed data entry | | Some concerns about missing AuSCR data; strong support for using existing registry data without extra collection burden | Complementary – Divergent – Appreciation for data + varying concerns about quality |

| Theme/sub-theme | Workshop observations and/or documentation* | Post-workshop survey | Support Activity Log | Interviews | Triangulation |
|---|---|---|---|---|---|
| Action plan characteristics - feasibility of prioritized indicators and strategies | Observed focus towards easier, task-based strategies being chosen | 90% respondents confident that the action plans could be implemented locally | Documented focus on some indicators more so than others | Several strategies seen as too complex for timeframe; selecting too many indicators was detrimental to implementation | Complementary – Divergent – feasible strategies inspired confidence + trade-off between practicality and ambition |
| Recipients | | | | | |
| Importance of multidisciplinary involvement | Multidisciplinary attendance strengthened robust discussions; Action plan developed by few staff at some hospitals; Limited representation from medical team and emergency department | Workload and rotation challenges identified in wider team engagement | | Engagement dependent on existing relationships; implementation of certain indicators required particular discipline involvement (e.g. emergency department for thrombosis provision) | Complementary |
| Context | | | | | |
| Organizational context | Pre-survey and challenges discussed during workshops highlighted limitations due to wider organizational factors | Alignment with other quality improvement initiatives beneficial | | Completing organizational challenges e.g new electronic medical record system; generalist stroke teams; change fatigue noted at some sites | Complementary |
| Management and leadership support | Limited medical leadership observed during workshops; Some indicators avoided due to limited medical-buy in even through performance gap | | | Insufficient resources/time challenged implementation; important to embed into continuous improvement | Complementary |

*includes attendance records, action plans
